# Models to predict length of stay in the emergency department: a systematic literature review and appraisal

**DOI:** 10.1186/s12873-024-00965-4

**Published:** 2024-04-04

**Authors:** Raheleh Mahboub Farimani, Hesam Karim, Alireza Atashi, Fariba Tohidinezhad, Kambiz Bahaadini, Ameen Abu-Hanna, Saeid Eslami

**Affiliations:** 1 Department of Medical Informatics, Kerman University of Medical Sciences, Kerman, Iran; 2 Department of Health Information Management, Faculty of Allied Medical Sciences, Tehran University of Medical Sciences, Tehran, Iran; 3 E-Health Department, Virtual School, Tehran University of Medical Sciences, Tehran, Iran; 4 Department of Medical Informatics, Faculty of Medicine, Mashhad University of Medical Sciences, Mashhad, Iran; 5 Medical Informatics, UMC Location University of Amsterdam, Meibergdreef, Amsterdam, The Netherlands; 6 Amsterdam Public Health, Amsterdam, The Netherlands; 7 Pharmaceutical Research Center, School of Pharmacy, Mashhad University of Medical Sciences, Mashhad, Iran

**Keywords:** Emergency department utilization, Length of stay, Crowding, Prediction models

## Abstract

**Introduction:**

Prolonged Length of Stay (LOS) in ED (Emergency Department) has been associated with poor clinical outcomes. Prediction of ED LOS may help optimize resource utilization, clinical management, and benchmarking. This study aims to systematically review models for predicting ED LOS and to assess the reporting and methodological quality about these models.

**Methods:**

The online database PubMed, Scopus, and Web of Science (10 Sep 2023) was searched for English language articles that reported prediction models of LOS in ED. Identified titles and abstracts were independently screened by two reviewers. All original papers describing either development (with or without internal validation) or external validation of a prediction model for LOS in ED were included.

**Results:**

Of 12,193 uniquely identified articles, 34 studies were included (29 describe the development of new models and five describe the validation of existing models). Different statistical and machine learning methods were applied to the papers. On the 39-point reporting score and 11-point methodological quality score, the highest reporting scores for development and validation studies were 39 and 8, respectively.

**Conclusion:**

Various studies on prediction models for ED LOS were published but they are fairly heterogeneous and suffer from methodological and reporting issues. Model development studies were associated with a poor to a fair level of methodological quality in terms of the predictor selection approach, the sample size, reproducibility of the results, missing imputation technique, and avoiding dichotomizing continuous variables. Moreover, it is recommended that future investigators use the confirmed checklist to improve the quality of reporting.

## Introduction

Overcrowding in the Emergency Department (ED) is an important worldwide problem [1–3] and it has received considerable international attention in recent years [4–8]. Rising demand for ED services and relative shortage of hospital beds are major causes of ED crowding and longer waiting times [4]. Length of Stay (LOS) in ED is usually defined as the time from patient registration in ED to patient discharge or transfer to another facility, or ward [2, 9]. ED LOS is perceived as an important component of ED overcrowding [7, 9] and a quality indicator for ED throughput [6].

Longer LOS in ED had poor clinical outcomes such as increased mortality/morbidity [7] and complication rates, decreased quality of care [1, 2] and patient satisfaction, ambulance diversion, and higher levels of recurrent ED crowding [2, 3]. Thus, LOS is an important measure of treatment timeliness when correcting for the severity of illness, patient safety, patient satisfaction, and quality of care in ED [2, 6, 8, 9]. Predicting length of stay is important in clinical and informatics research [10] and important to improve ED care and efficiency [3, 11]. The model’s predicted ED LOS may provide useful information for physicians or patients to better anticipate an individual’s LOS and to help the administrative level plan its staffing policy [12]. Additionally, the development of a prediction tool could assist in bed management and patient flow through ED and hospitals [13].

Many studies have been conducted to develop ED LOS prediction models. However, to the best of our knowledge, no previous systematic literature review has summarized these studies. Given the lack of evidence, additional research is needed to explore the related studies in this area and to address this knowledge gap. Considering recent evidence demonstrating the limited implementation and thus limited impact of hospital policies to improve patient flow through the ED is important [10, 11].

This study aims to systematically review and appraise the reporting and methodological quality of all development (with or without internal validation) and external validation studies describing a model aimed at predicting LOS in ED. It also provides recommendations for improving their reporting a prediction model for ED LOS.

## Methods

### Search strategy

We searched the PubMed (Medline), Scopus, and Web of Science databases for journal articles based on keywords in all fields until 10 September 2023, using the following query: ("length of stay") AND (emergency OR urgent) AND (prognostic OR prognosis OR predict*). All references were imported into the literature management program EndNote. All results were screened for relevance against our inclusion and exclusion criteria.

### Inclusion and exclusion criteria

All original papers were included if they have described either the development (with or without internal validation) or external validation of a prediction model for LOS in emergency department patients. All duplicate articles, conference abstracts, and reviews were excluded. Only English articles were included. The review follows the 2020 Preferred Reporting Items for Systematic Reviews and Meta-Analyses (PRISMA) guidelines recommended by the Cochrane Handbook for Systematic Reviews of Interventions [13].

### Selection of studies

**Fig. 1:**
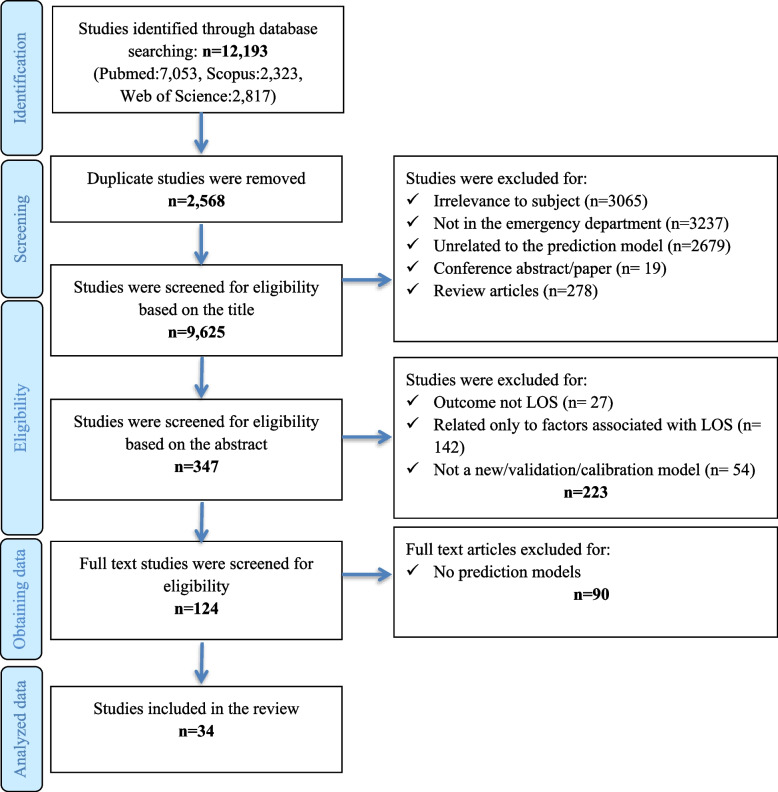
PRISMA flow diagram of the study screening process

Two reviewers (H. K and R. F) independently screened the titles and abstracts using Rayyan^1^ research tool. Rayyan provides cooperative work on the systematics review and easy to orders articles and extracts data for blinded screening and automatic removal of duplicates. The results were compared and discussed until a consensus was reached. Discrepancies between the two reviewers were resolved by consensus involving a third reviewer (S. E). Figure 1 shows the search flowchart.

### Assessment of methodological and reporting quality

We used a checklist developed for critical appraisal and data extraction for systematic reviews of prediction modeling studies (CHARMS) [14]. This consists of eleven domains, each containing several (one to six) key items, resulting in a total of 32 key items [14]. We extended this checklist with three additional items taken from a scoring framework for assessing the quality of reporting in prediction model development studies [12] (Table 1). The total number of included key items was 39 for 12 different domains.

**Table 1:** Adopted domains and (key) items of the used CHARMS [15] checklist accompanied by the reporting- and methodological score per item

**All studies [Reference]**																		
**Studies**	Lee S. et al. (2023) [16]	Zeleke AJ. et al. (2023) [17]	Lee H. et al. (2023) [18]	Kadri F. et al. (2023) [19]	Lee KS. et al. (2022) [20]	Srivastava S. et al. (2022) [21]	Etu EE. et al. (2022) [22]	Chang YH. et al. (2022) [23]	d'Etienne JP. et al. (2021) [24]	Laher AE. et al. (2021) [25]	Bacchi S. et al. (2020) [15]	Sweeny A. et al. (2020) [26]	Sricharoen P. et al. (2020) [27]	Rahman MA. et al. (2020) [28]	Curiati PK. et al. (2020) [29]	Chen C-H. et al. (2020) [30]	Street, M. et al. (2018) [31]	Gill, S. D. et al. (2018) [32]
**Key items**																		
**Source of data**																		
Source of data (e.g., cohort, case–control, randomized trial participants, or registry data)^a^	y	y	y	y	y	y	y	y	y	y	y	y	y	y	y	y	y	y
**Participants**																		
Participant eligibility and recruitment method (e.g., consecutive participants, location, number of centers, setting, country, inclusion and exclusion criteria)^a^	y	y	y	y	y	y	y	y	p	y	y	y	y	p	y	y	y	y
Participant description	y	y	y	y	y	y	y	y	y	y	y	y	y	y	y	y	y	y
Study dates	y	y	y	y	y	y	y	y	y	y	y	y	y	y	y	y	y	y
**Outcome(s) to be predicted**																		
Definition and method for measurement of outcome	y	y	y	y	y	y	y	y	y	y	y	y	y	y	y	y	p	p
Was the same outcome definition (and measurement method) used in all patients?	y	y	y	y	y	y	y	y	y	y	y	y	y	y	y	y	y	y
Type of outcome (e.g., single or combined endpoints)	y	y	y	y	y	y	y	y	y	y	y	y	y	y	y	y	y	y
Where candidate predictors part of the outcome (e.g., in panel or consensus diagnosis)?	n	n	n	y	n	n	n	y	n	n	n	n	n	n	n	n	n	n
**Candidate predictor**																		
Number and type of predictors (e.g., demographics, patient history, physical examination, additional testing, disease characteristics)	y	y	y	y	y	y	y	y	y	y	y	y	p	p	p	y	y	y
Definition and method for measurement of candidate predictors	y	y	y	y	y	y	y	y	y	y	y	y	y	y	y	y	y	y
Timing of predictor measurement (e.g., at patient presentation, at diagnosis, at treatment initiation)	y	y	y	y	y	y	y	y	y	y	y	y	p	y	p	y	y	y
Handling of predictors in the modeling (e.g., continuous, linear, non-linear transformations or categorized)	y	y	y	y	y	y	y	y	n	n	y	n	n	n	n	n	n	n
**Sample size**																		
Number of participants and number of outcomes/events	y	y	y	y	y	y	y	y	y	y	y	y	y	y	y	y	y	y
Number of outcomes/events in relation to the number of candidate predictors (Events Per Variable)^a^	y	y	y	y	y	y	y	y	y	y	y	y	y	y	y	y	y	y
**Missing data**																		
Number of participants with any missing value (include predictors and outcomes)	n	y	n	n	n	y	n	y	n	n	n	n	n	n	n	n	n	n
Number of participants with missing data for each predictor	n	n	n	n	n	n	n	n	n	n	n	n	n	n	n	n	n	n
Handling of missing data (e.g., complete-case analysis, imputation, or other methods)	n	n	n	y	n	n	y	n	n	n	n	n	n	y	n	n	n	n
**Model development**																		
Modeling method (e.g., logistic, survival or machine learning techniques)	y	y	y	y	y	y	y	y	y	y	y	y	y	y	y	y	y	y
Modelling assumptions satisfied	y	y	y	y	y	y	y	y	y	y	y	y	y	y	y	y	y	y
Method for selection of predictors for inclusion in multivariable modeling (e.g., all candidate predictors, pre-selection based on unadjusted association with the outcome)	y	y	y	y	y	y	y	y	y	y	y	y	y	y	y	y	y	y
Initial predictors/variables are reported such that the results are reproducible^b^	y	y	y	y	y	y	y	y	y	y	y	y	y	y	y	y	y	y
Method for selection of predictors during multivariable modeling (e.g., full model approach, backward or forward selection) and criteria used (e.g., *p*-value, Akaike Information Criterion)	y	y	y	y	y	y	y	y	y	y	n	n	y	y	y	n	y	y
Shrinkage of predictor weights or regression coefficients (e.g., no shrinkage, uniform shrinkage, penalized estimation)	n	n	n	n	n	n	n	n	n	n	n	n	n	n	y	n	n	n
Reporting of model derivation and calibration process is sufficient for the results to be reproduced^b^	n	y	y	n	n	n	n	n	n	n	n	n	n	y	y	n	n	n
**Handling specific patient subgroups 3**																		
Readmissions^a^	n	n	n	n	n	n	n	n	n	n	n	n	n	n	n	n	n	n
Transfers^a^	n	n	n	n	n	n	n	n	n	n	n	n	n	n	n	n	n	n
Non-survivors^a^	n	n	n	n	n	n	n	n	n	n	n	n	n	n	n	n	n	n
Cardiac surgery^a^	n	n	n	n	n	n	n	n	n	n	n	n	y	n	n	n	n	n
**Model performance**																		
Calibration (calibration plot, calibration slope, Hosmer–Lemeshow test) and Discrimination	n	y	y	n	n	n	n	n	n	n	n	n	n	n	y	n	y	n
(C-statistic, D-statistic, log-rank) measures with confidence intervals	n	n	n	n	y	y	n	y	y	y	n	y	n	n	y	n	n	n
Classification measures (e.g., sensitivity, specificity, predictive values, net reclassification improvement) and whether a-priori cut points were used	y	y	n	n	n	n	y	y	y	n	y	n	y	y	y	n	n	n
**Model evaluation**																		
Method used for testing model performance: development dataset only (random split of data, resampling methods e.g. bootstrap or cross-validation, none) or separate External validation (e.g. temporal, geographical, different setting, different investigators)^a^	y	y	y	y	n	n	y	y	y	n	y	n	y	y	y	y	y	y
In case of poor validation, whether model was adjusted or updated (e.g., intercept recalibrated, predictor effects adjusted, or new predictors added)	n	n	n	n	n	n	n	n	n	n	n	n	n	n	n	n	n	n
**Publication of the developed models (Results)**																		
Final and other multivariable models (e.g., basic, extended, simplified) presented, including predictor weights or regression coefficients, intercept, baseline survival, model performance measures (with standard errors or confidence intervals)^a^	y	y	y	y	y	y	y	y	y	y	y	y	y	y	y	y	y	y
Any alternative presentation of the final prediction models, e.g., sum score, monogram, score chart, predictions for specific risk subgroups with performance	n	n	n	n	n	n	n	n	n	n	n	n	n	n	n	n	n	n
Comparison of the distribution of predictors (including missing data) for development and validation datasets	n	n	n	n	n	n	n	n	n	n	n	n	n	p	y	n	y	n
**Interpretation and discussion**																		
Interpretation of presented models (confirmatory, i.e., model useful for practice versus exploratory, i.e., more research needed)	y	y	y	y	y	y	y	y	y	y	y	y	y	y	y	y	y	y
Comparison with other studies, discussion of generalizability	y	y	y	n	y	y	y	p	y	y	y	y	y	y	y	y	y	y
Strengths, weakness, limitations and future challenges	y	y	p	p	y	p	p	y	p	p	y	y	p	y	y	p	p	p
**Methodological quality items**																		
Study consists of a cohort study or registry instead of a randomized design (source of data)	y	y	y	y	y	y	y	y	y	y	y	y	y	y	y	y	y	y
Study consists of a prospective study design (source of data)	n	n	n	n	n	n	n	n	n	n	n	n	n	n	n	n	n	n
Patients are excluded based on outcome variable (participants)	n	n	n	n	n	n	n	n	n	n	n	n	n	n	n	n	n	n
Selective inclusion based on data availability took place (participants)	n	n	n	n	n	n	n	n	n	n	n	n	n	n	n	n	n	n
Sample size (n) in development set is sufficient relative to the number of variables in the final model (sample size)	y	y	y	y	y	y	y	y	y	y	y	y	y	y	y	y	y	y
Specific treatment for this subgroup took place: readmissions	n	n	n	n	n	n	n	n	n	n	n	n	n	n	n	n	n	n
Specific treatment for this subgroup took place: transfers	n	n	n	n	n	n	n	n	n	n	n	n	n	n	n	n	n	n
Specific treatment for this subgroup took place: non-survivors	n	n	n	n	n	n	n	n	n	n	n	n	n	n	n	n	n	n
Specific treatment for this subgroup took place: cardiac surgery	n	n	n	n	n	n	n	n	n	n	n	n	y	n	n	n	n	n
Validation took place using an independent validation dataset (model evaluation)	n	n	n	n	n	n	n	y	y	n	n	n	n	n	y	n	n	n
Model is reproducible (results of the developed models)	y	y	y	y	y	y	y	y	y	y	y	y	y	y	y	y	n	n
**Total score**																		
Reporting score	54	60	55	53	52	53	55	61	54	49	52	48	53	55	62	47	50	46
Reporting score (%)	54	60	55	53	52	53	55	61	54	49	52	48	53	55	62	47	50	46
Methodological score	6	6	6	6	6	6	6	8	8	6	6	6	8	6	8	6	4	4
Methodological score (%)	27	27	27	27	27	27	27	36	36	27	27	27	36	27	36	27	18	18

**All studies [Reference]**																		
**Studies**	Zhu, T. et al. (2017) [33]	Chaou C-H. et al. (2017) [34]	Mark B. Warren (2016) [35]	Prisk D. et al. (2016) [36]	Launay CP. Et al (2015) [37]	Stephens R. et al. (2014) [38]	Casalino, E. et al. (2014) [39]	Green N. et al. (2012) [40]	van der Linden C. et al. (2012) [41]	Nejtek V. A. et al. (2011) [42]	Ru Ding (2010) [43]	Chi, C. H. et al. (2006) [44]	Walsh P. et al. (2004) [45]	Tanabe P. et al. (2004) [46]	Jimenez, J. G. et al. (2003) [47]	Tandberg D. et al. (1994) [48]	**Total score key item**	**Percentage of score key item (%)**
**Key items**																		
**Source of data**																		
Source of data (e.g., cohort, case–control, randomized trial participants, or registry data)^a^	y	y	y	y	y	y	y	y	y	y	y	y	y	y	p	y	67	99
**Participants**																		
Participant eligibility and recruitment method (e.g., consecutive participants, location, number of centers, setting, country, inclusion and exclusion criteria)^a^	y	y	y	y	y	y	p	p	y	p	p	y	n	p	p	p	57	84
Participant description	y	y	p	y	y	y	p	y	y	y	n	y	n	n	n	n	56	82
Study dates	y	y	y	y	y	y	y	y	p	y	y	y	p	y	y	y	66	97
**Outcome(s) to be predicted**																		
Definition and method for measurement of outcome	y	y	p	y	y	p	y	y	y	n	y	n	n	y	n	y	56	82
Was the same outcome definition (and measurement method) used in all patients?	y	y	y	y	y	y	y	y	y	y	y	y	y	y	y	y	68	100
Type of outcome (e.g., single or combined endpoints)	y	y	y	y	y	y	y	y	y	y	y	n	y	y	y	y	66	97
Where candidate predictors part of the outcome (e.g., in panel or consensus diagnosis)?	n	n	n	n	y	y	n	n	n	n	n	n	n	n	n	n	8	12
**Candidate predictor**																		
Number and type of predictors (e.g., demographics, patient history, physical examination, additional testing, disease characteristics)	y	y	y	p	y	y	y	y	y	y	y	y	y	y	y	y	64	94
Definition and method for measurement of candidate predictors	y	y	y	y	y	y	y	y	y	y	y	y	y	y	y	y	68	100
Timing of predictor measurement (e.g., at patient presentation, at diagnosis, at treatment initiation)	y	y	y	y	y	y	y	y	y	y	y	y	y	y	y	y	66	97
Handling of predictors in the modeling (e.g., continuous, linear, non-linear transformations or categorized)	n	y	y	p	y	y	p	p	n	p	y	y	n	n	n	n	44	65
**Sample size**																		
Number of participants and number of outcomes/events	y	y	y	y	y	y	y	y	y	y	y	y	y	y	y	y	68	100
Number of outcomes/events in relation to the number of candidate predictors (Events Per Variable)^a^	y	y	y	y	y	y	y	y	y	y	y	y	y	y	y	y	68	100
**Missing data**																		
Number of participants with any missing value (include predictors and outcomes)	n	n	n	n	n	n	n	n	n	y	y	n	n	n	y	n	12	18
Number of participants with missing data for each predictor	n	n	n	n	n	n	n	n	n	y	y	n	n	n	y	n	6	9
Handling of missing data (e.g., complete-case analysis, imputation, or other methods)	n	p	n	n	n	y	n	n	n	n	y	n	n	n	y	n	13	19
**Model development**																		
Modeling method (e.g., logistic, survival or machine learning techniques)	y	y	y	y	y	y	y	p	p	y	y	p	y	p	p	y	63	93
Modelling assumptions satisfied	y	y	y	y	y	y	y	y	p	y	y	y	y	y	y	y	67	98
Method for selection of predictors for inclusion in multivariable modeling (e.g., all candidate predictors, pre-selection based on unadjusted association with the outcome)	n	y	y	y	y	y	n	n	y	y	y	n	y	n	n	n	54	79
Initial predictors/variables are reported such that the results are reproducible^b^	n	y	y	p	y	y	y	y	y	n	n	n	y	n	n	n	53	78
Method for selection of predictors during multivariable modeling (e.g., full model approach, backward or forward selection) and criteria used (e.g., *p*-value, Akaike Information Criterion)	n	y	y	y	y	y	n	n	y	y	y	n	y	n	n	n	48	71
Shrinkage of predictor weights or regression coefficients (e.g., no shrinkage, uniform shrinkage, penalized estimation)	n	n	n	n	n	n	n	n	n	n	n	n	n	n	n	n	2	3
Reporting of model derivation and calibration process is sufficient for the results to be reproduced^b^	n	y	n	n	n	n	n	n	n	n	n	n	y	n	n	y	14	21
**Handling specific patient subgroups 3**																		
Readmissions^a^	n	n	n	n	n	n	n	n	n	n	n	n	n	n	n	n	0	0
Transfers^a^	n	n	n	n	n	n	n	n	n	n	n	n	n	n	n	n	0	0
Non-survivors^a^	n	n	n	n	n	n	n	n	n	n	n	n	n	n	n	n	0	0
Cardiac surgery^a^	n	n	n	n	n	n	n	n	n	n	n	n	n	n	n	n	2	3
**Model performance**																		
Calibration (calibration plot, calibration slope, Hosmer–Lemeshow test) and Discrimination	n	y	y	n	n	y	n	y	y	y	y	n	y	y	n	y	28	41
(C-statistic, D-statistic, log-rank) measures with confidence intervals	y	n	n	n	n	y	p	n	n	p	n	p	n	n	p	n	22	32
Classification measures (e.g., sensitivity, specificity, predictive values, net reclassification improvement) and whether a-priori cut points were used	n	n	n	n	y	n	n	n	n	n	n	n	y	n	n	n	22	32
**Model evaluation**																		
Method used for testing model performance: development dataset only (random split of data, resampling methods e.g. bootstrap or cross-validation, none) or separate External validation (e.g. temporal, geographical, different setting, different investigators)^a^	n	n	n	n	n	y	y	n	y	p	y	n	y	y	n	y	43	63
In case of poor validation, whether model was adjusted or updated (e.g., intercept recalibrated, predictor effects adjusted, or new predictors added)	n	n	n	n	n	n	n	n	n	n	n	n	n	n	n	n	0	0
**Publication of the developed models (Results)**																		
Final and other multivariable models (e.g., basic, extended, simplified) presented, including predictor weights or regression coefficients, intercept, baseline survival, model performance measures (with standard errors or confidence intervals)^a^	y	y	p	p	y	y	y	p	p	y	y	y	y	y	p	y	63	93
Any alternative presentation of the final prediction models, e.g., sum score, monogram, score chart, predictions for specific risk subgroups with performance	n	n	n	n	n	n	n	n	n	n	n	n	n	n	n	n	0	0
Comparison of the distribution of predictors (including missing data) for development and validation datasets	y	y	n	n	n	y	y	y	p	y	y	y	n	n	p	n	23	34
**Interpretation and discussion**																		
Interpretation of presented models (confirmatory, i.e., model useful for practice versus exploratory, i.e., more research needed)	y	y	y	y	y	y	y	y	y	y	y	y	y	y	y	y	68	100
Comparison with other studies, discussion of generalizability	y	y	n	y	y	y	n	y	n	n	n	n	n	y	n	n	47	69
Strengths, weakness, limitations and future challenges	p	y	y	y	p	p	y	y	p	p	n	y	y	y	y	y	51	75
**Methodological quality items**																		
Study consists of a cohort study or registry instead of a randomized design (source of data)	y	y	y	y	y	y	y	y	y	y	y	y	y	y	y	y	68	100
Study consists of a prospective study design (source of data)	n	n	n	n	y	n	y	y	n	n	n	y	n	n	n	y	10	14
Patients are excluded based on outcome variable (participants)	n	n	n	n	n	n	n	n	n	n	n	n	n	n	n	n	0	0
Selective inclusion based on data availability took place (participants)	n	n	n	n	n	n	n	n	n	n	n	n	n	n	n	n	0	0
s Sample size (n) in development set is sufficient relative to the number of variables in the final model (sample size)	y	y	y	y	y	y	y	y	y	y	y	y	y	y	y	y	68	100
Specific treatment for this subgroup took place: readmissions	n	n	n	n	n	n	n	n	n	n	n	n	n	n	n	n	0	0
Specific treatment for this subgroup took place: transfers	n	n	n	n	n	n	n	n	n	n	n	n	n	n	n	n	0	0
Specific treatment for this subgroup took place: non-survivors	n	n	n	n	n	n	n	n	n	n	y	n	n	n	n	n	2	3
Specific treatment for this subgroup took place: cardiac surgery	n	n	n	n	n	n	n	n	n	n	n	n	n	n	n	n	2	3
Validation took place using an independent validation dataset (model evaluation)	n	n	n	n	n	y	n	n	n	n	n	n	y	n	n	y	12	18
Model is reproducible (results of the developed models)	y	y	n	y	y	y	y	n	n	n	n	n	y	n	n	n	46	68
**Total score**																		
Reporting score	45	57	45	46	55	62	48	46	44	49	53	40	49	40	38	45	1731	
Reporting score (%)	45	57	45	46	55	62	48	46	44	49	53	40	49	40	38	45	50	
Methodological score	6	6	4	6	8	8	8	6	4	4	6	6	8	4	4	8	208	
Methodological score (%)	27	27	18	27	36	36	36	27	18	18	27	27	36	18	18	36	28	

^a^One or more methodological scores are given to this item

^b^Additional items were added to the checklist from a scoring framework developed for reviewing models to predict mortality in very premature infants [14]

We extracted 11 items from the literature to evaluate the methodological quality of model development studies [12, 14, 49, 50] (Table 2).

**Table 2:** Summary of exclusion used to include ED admissions for model development and/or model validation. Information on predictor variables included and/or predictor variables applied in the model which is validated by the included studies

**Reviewed studies (references)**																	
	Lee S. et al. (2023) [16]	Zeleke AJ. et al. (2023) [17]	Lee H. et al. (2023) [18]	Kadri F. et al. (2023) [19]	Lee KS. et al. (2022) [20]	Srivastava S. et al. (2022) [21]	Etu EE. et al. (2022) [22]	Chang YH. et al. (2022) [23]	d'Etienne JP. et al. (2021) [24]	Laher AE. et al. (2021) [25]	Bacchi S. et al. (2020) [15]	Sweeny A. et al. (2020) [26]	Sricharoen P. et al. (2020) [27]	Rahman MA. et al. (2020) [28]	Curiati PK. et al. (2020) [29]	Chen C-H. et al. (2020) [30]	Street, M. et al. (2018) [31]
**Exclusion criteria (participants)**																	
Left without being seen		y						y									
Left against medical advice		y						y								y	
Eloped																y	
Ed deaths		y						y				y	y				
Outpatient																	
Registration errors, incomplete data, missing data					y	y		y	y				y				
Elsewhere and not treated in the study EDs								y									
Visits with multiple missing time, invalid discharge times													y				
Other than trauma patients								y								y	
Other than mental patients																	
Based on age	y				y	y	y	y		y		y	y		y	y	y
Refused research consent										y							
**Predictors included in the model developed (candidate predictors)**																	
Number of continuous variables		1		9	2	1		18		8	2	3	10	1	7	2	3
Number of categorical variables	5	6	19		9	8	6	5	14	4		4	4	6		3	9
Number of binary variables				3													
Categorizing continuous variables (y/n)	y	y	y		y		y		y	y		Y	y				
Categorizing all continuous variables (y/n)	y	y	y														
Preoperative risk factors (y/n)															y		y
Intra (or post)operative factors (y/n)									y	y					y	y	
**Variables included as covariate**																	
Age	y	y	y	y	y	y	y	y	y	y			y	y	y	y	y
Gender	y	y	y	y	y	y	y	y	y	y		y	y	y	y	y	
Race							y		y	y							
Marital status									y								
Mode of Arrival		y										y		y			
Admission type																	
Current diagnosis		y		y		y	y							y			
Mental state														y			
Smoking																	
Blood alcohol																	
Number of ED admissions								y									y
Patient disposition						y											
Chief complaint									y								
Acuity levels			y													y	
Pharmacotrapy																	
ED occupancy																	
Medicine bed																	
Day of week												y		y			
Time of day			y	y								y		y		y	
Month of visit																	
Setting						y											
Disease type												y	y				
Death	y									y							
Observation with LOS > 24 h																	
Oxygen saturation after initial treatment							y			y						y	
Increased work of breathing																	
Tachycardia on entry																	
Wheezing only																	
Dehydration																	
Unit type																	
Insurance type	y				y	y											
Region						y											
Treatment area																	
Admission time					y												
Triage category		y	y		y				y			y		y		y	y
Time to imaging request																	
Time to bed request																	
Time to pathology request																	
Time to clinician allocation														y			
Time to handover																	
Diagnosis group		y						y									
Month of arrival																	
Type of image request																	
Clinician group																	
Involvement by PART^a^																	
Arrival time			y									y				y	
Day of arrival												y					
Hospital occupancy at arrival																	
non-FT^b^ occupancy at arrival																	
FT occupancy at arrival																	
non-FT waiting to be seen at arrival																	
non-FT bed requested at arrival																	
FT waiting to be seen at arrival																	
FT bed requested at arrival																	
Discharge destination												y					
Type of usual accommodation																	
Compensable status																	
Source of referral									y					y			
Required imaging																	y
Seen by doctor(time)																	y
ED overcrowding									y								y
Access block																	y
Required pathology																	y
ED arrival overnight					y												y
Arrival by ambulance								y									y
Lived in RACF^c^																	y
Required imaging and arrived overnight																	y
Model assumptions tested																	
X square	y				y	y				y		y					y
ANOVA (one way)			y			y	y										
Kappa statistic																	
Kaplan meier																	
T test			y													y	
Fisher										y							
Mann–Whitney																	y
Kruskal–Wallis																	
Hosmer- Lemeshow			y														

**Reviewed studies (references)**																	
	Gill, S. D. et al. (2018) [32]	Zhu, T. et al. (2017) [33]	Chung-Hsien Chaou. et al. (2017) [34]	Mark B. Warren (2016) [35]	Prisk D. et al. (2016) [36]	Launay CP. Et al (2015) [37]	Stephens R. et al. (2014) [38]	Casalino, E. et al. (2014) [39]	Green N. et al. (2012) [40]	van der Linden C. et al. (2012) [41]	Nejtek V. A. et al. (2011) [42]	Ru Ding (2010) [43]	Chi, C. H. et al. (2006) [44]	Walsh P. et al. (2004) [45]	Tanabe P. et al. (2004) [46]	Jimenez, J. G. et al. (2003) [47]	Tandberg D. et al. (1994) [48]
**Exclusion criteria (participants)**																	
Left without being seen	y						y			y		y				y	
Left against medical advice							y					y					
Eloped							y										
Ed deaths												y					
Outpatient											y						
Registration errors, incomplete data, missing data		y	y				y					y				y	
Elsewhere and not treated in the study EDs												y					
Visits with multiple missing time, invalid discharge times												y					
Other than trauma patients													y				
Other than mental patients							y										
Based on age								y	y		y		y				
Refused research consent				y													
**Predictors included in the model developed (candidate predictors)**																	
Number of continuous variables		4	4	1		11	1		1		1	1	1		1		
Number of categorical variables		3	3	9	7	1	10		3		8	3	3		0		
Number of binary variables		1	1	5			1		1		1	1	4				
Categorizing continuous variables (y/n)		y		y	y		y		y		y	y	y			y	
Categorizing all continuous variables (y/n)		y		y	y		y		y			y	y			y	
Preoperative risk factors (y/n)								y									
Intra (or post)operative factors (y/n)																	
**Variables included as covariate**																	
Age	y	y	y	y	y	y	y	y			y	y	y		y		
Gender	y	y	y	y		y	y	y			y	y	y		y		
Race				y			y				y						
Marital status											y						
Mode of Arrival	y	y	y	y			y	y				y					
Admission type													y				
Current diagnosis					y		y				y						
Mental state				y							y						
Smoking														y			
Blood alcohol				y													
Number of ED admissions											y						
Patient disposition				y	y		y				y		y				
Chief complaint							y					y					
Acuity levels			y				y	y				y	y	y			
Pharmacotrapy				y							y						
ED occupancy				y								y					
Medicine bed												y					
Day of week			y	y			y					y					
Time of day		y	y	y								y					
Month of visit			y	y													
Setting													y				
Disease type													y				
Death													y				
Observation with LOS > 24 h			y										y				
Oxygen saturation after initial treatment														y			
Increased work of breathing														y			
Tachycardia on entry														y			
Wheezing only														y			
Dehydration														y			
Unit type							y										
Insurance type				y			y				y	y					
Region		y															
Treatment area		y															
Admission time		y															
Triage category	y	y			y												
Time to imaging request	y																
Time to bed request	y																
Time to pathology request	y																
Time to clinician allocation	y																
Time to handover	y																
Diagnosis group	y																
Month of arrival	y																
Type of image request	y																
Clinician group	y				y												
Involvement by PART^a^	y																
Arrival time	y	y															
Day of arrival	y																
Hospital occupancy at arrival	y																
non-FT^b^ occupancy at arrival	y																
FT occupancy at arrival	y																
non-FT waiting to be seen at arrival	y																
non-FT bed requested at arrival	y																
FT waiting to be seen at arrival	y																
FT bed requested at arrival	y																
Discharge destination	y																
Type of usual accommodation	y																
Compensable status	y																
Source of referral	y																
Required imaging																	
Seen by doctor(time)																	
ED overcrowding																	
Access block																	
Required pathology																	
ED arrival overnight																	
Arrival by ambulance																	
Lived in RACF^c^																	
Required imaging and arrived overnight																	
Model assumptions tested																	
X square			y		y	y	y	y	y		y		y			y	
ANOVA (one way)		y											y				
Kappa statistic														y			
Kaplan meier			y											y			
T test					y					y					y		y
Fisher								y								y	
Mann–Whitney			y					y								y	
Kruskal–Wallis			y					y								y	
Hosmer- Lemeshow				y													

^a^Planning and Referral Team

^b^Fast Track

^c^Residential Aged Care Facility

Each key item was rated as ‘yes’, ‘partly’, or ‘not’ for the reporting as well as for the methodological quality, with a respective score of 2, 1, or 0. We summarized these results to rate the reporting and methodological quality of the model development studies. Table 2 describes the extracted data items to quantify each particular domain of the checklist.

## Results

### Search strategy

Online searching resulted in 12,193 articles. Initial screening of titles and abstracts rendered 124 articles for full-text review. Based on the full-text review, 90 articles were excluded because they focused on factors associated with ED LOS, or no prediction model was reported. As shown in Table 3, 34 articles were included for full-text analysis and data extraction. In total, 29 models were developed [15–37, 39, 42, 43, 45, 47, 48] and five studies [40, 41, 44, 46, 47] described the validation of the Emergency Severity Index (ESI), Canadian emergency department Triage and Acuity Scale (CTAS), or ENP-stream models.

**Table 3:** Characteristics of the selected studies for the systematic review

N	Author	Year	Journal	Country and ED setting	EDLOS Cut-Off
1	Lee S [16]	2023	Personalized Medicine	1 U.S	(≤ 24 h, ≤ 48 h, ≤ 4 days, ≤ 7 days)
2	Zeleke AJ [17]	2023	Frontiers in Artificial Intelligence	1 Italy	≥ 6 h
3	Lee H [18]	2023	Nursing Open	1 Korea	(≤ 6, > 6)h
4	Kadri F [19]	2023	Ambient Intelligence and Humanized Computing	1 France	(≤ 120, 120–210, 210–300, 300–480, > 480)min
5	Lee K.S [20]	2022	BMC Emergency Medicine	1 Korea	(< 6, ≥ 6)h
6	Srivastava S [21]	2022	Journal of Hypertension	1 U.S	Continious
7	Etu EE [22]	2022	IEEE Access	1 U.S	Continious
8	Chang YH [23]	2022	BMC Emergency Medicine	1 Taiwan	< 4 h, ≥ 4 h
9	d'Etienne JP [24]	2021	Am J Emerg Med	1 U.S	(6, 8, 12, 16, 23) h
10	Laher AE [25]	2021	PloS one	1 South Africa	(< 7, ≥ 7)days
11	Bacchi S [15]	2020	Internal and Emergency Medicine	1 Australia	(< 2, ≥ 2)days
12	Sweeny A [26]	2020	Internal Medicine J	1 Australia	(> 4, > 6, > 8)h
13	Sricharoen P [21]	2020	Medicina	1 Thailand	Continuous
14	Rahman MA [28]	2020	Emergency Medicine Australasia	1 Australia	< 4 h, ≥ 4 h
15	Curiati PK [29]	2020	Annals of Emergency Medicine	1 Brazil	Continuous
16	Chen C-H [30]	2020	The American Journal of Emergency Medicine	1 Taiwan	< 6 h, ≥ 6 h
17	Street, M [31]	2018	European Journal of Emergency Medicine	1 Australia	> 4 h
18	Gill, S. D [32]	2018	Emergency Medicine Australasia	1 Australia	(0,50,100,150,200,250)min
19	Zhu, T [33]	2017	IEEE journal of biomedical and health informatics	1 China	(> 4 h, > 6 h, > 24 h, > 72 h, less than one week)
20	Chaou C-H [34]	2017	PloS one	1 China	Continuous
21	Warren M [35]	2016	Am J Emerg Med	1 U.S	< 8 h, ≥ 8 h
22	Prisk D [36]	2016	West J Emerg Med	1 New Zealand	Continuous
23	Launay CP [37]	2015	European Journal of Internal Medicine	1 France	Continuous
24	Stephens R [38]	2014	J Emerg Med	1 U.S	> 24 h
25	Casalino E [39]	2012	Emerg Med J	1 France	(< 160, ≥ 160, < 485, ≥ 485) min
26	Green N [40]	2012	Pediatr Emerg Care	1 U.S	Continuous
27	van der Linden C [41]	2012	Int Emerg Nurs	1 Netheland	Continious
28	Nejtek V. A [42]	2011	J Psychiatr Pract	1 U.S	(1–6, 7–12, 13–24, 25–48, 49–72, > 72)h
29	Ding R [43]	2010	Acad Emerg Med	4 U.S	Continious
30	Chi, C. H [44]	2006	J Formos Med Assoc	1 Taiwan	(< 6, 6–24, 24–48, > 48)h
31	Walsh P [45]	2004	Eur J Emerg Med	1 U.S	Continious
32	Tanabe P [46]	2004	J Emerg Nurs	1 U.S	Continious
33	Jimenez, J [47]	2003	Cjem	1 Andorra	Continious
34	Tandberg D [48]	1994	Ann Emerg Med	1 Mexico	Continious

*h* hour, *min* minute

### Assessment of methodological and reporting quality

#### Source of data

All studies used a cohort study design. A total of 28 studies were retrospective [15–20, 22–36, 40–43, 45–47] and four were prospective [37, 39, 44, 48]. One study used the case–control design [38] and one study used cross-sectional analysis [21].

#### Participants

Only one paper did not report the year of study [45]. The year of emergency admission for the rest of the studies ranged from 1989 [48] to 2022 [17, 29, 32]. The minimum and maximum duration of data collection was 2 months [15] and 4 years [16, 20], respectively. All studies were conducted in 13 countries of which 12 studies were performed in the United States [16, 21, 22, 24, 38, 40, 42, 43, 45, 46, 48, 51] and other studies done in The Netherlands [41], France[37, 39, 52], Taiwan [23, 30, 34, 44], Andorra [47], Australia [15, 26, 28, 31, 32], South Africa [25]), Thailand [27], Brazil [29], Korea [18, 20], New Zealand [36], Italy [17] and China [33]. Studies were conducted in general (*N* = 20) [15–17, 20–22, 24, 26–28, 30, 32, 33, 36, 41, 43, 45–48], mental (*N* = 3) [35, 38, 42], adult (*N* = 5) [18, 23, 25, 39, 44, 53], old people (*N* = 4) [26, 29, 31, 37] and pediatric (*N* = 2) EDs [40, 52]. All studies included all patients who were admitted in EDs during the period of their study and most of them extracted patient data from electronic patient databases. Table 3 shows the characteristics of the selected studies for the systematic review.

As shown in Table 1, eight studies [15, 18, 19, 28, 35, 45, 46, 48] had no specific exclusion/inclusion criteria and selected all patients who were admitted to EDs. There were different exclusion criteria in the rest of the studies. ED deaths and trauma or mental patients were excluded from 11 studies [17, 23, 26, 27, 31, 33, 38–40, 42–44]. Other studies excluded patients who left without being seen or without physician assessment [17, 23, 30, 32, 33, 38, 41, 43, 47], left after medical advice [23, 38, 43], eloped [23, 38] or those considered as outpatients [17, 23, 42]. Other exclusion criteria were: age restrictions [16, 20, 21, 26, 27, 29, 30, 37, 39, 40, 42, 44], ethnicity restriction [36], registration errors, incomplete or missing data [20, 21, 24, 27, 38, 43, 47], no confirmation of COVID-19 [22], treated elsewhere and not in the study EDs, and visits with multiple missing time or invalid discharge time [34, 43].

Only one study included patients who left the ED against medical advice (including discharge due to critical condition), who were transferred to another hospital, or were discharged from the ED after LOS > 24 h of observation, and/or died in the ED [44]. Other studies did not mention readmissions, transfer from or to another ED/Hospital, and patients who did not survive ED stay.

#### Outcome(s) to be predicted

Number of (primary and secondary) outcome variables in the included studies varied from one [16, 18, 19, 22, 23, 25, 27, 28, 30, 35–37, 44] to five [29]. Eighteen studies clearly defined outcome variable(s) [15–20, 22–25, 27, 31–33, 36–41, 46]. The others did not provide a clear definition for LOS. The lack of a unique definition for the LOS in ED might have led to different results. Seven studies defined ED LOS as a number of minutes (or hours) between a patient’s arrival/identification to ED and discharge [24, 27, 38–41, 46]. The primary outcome measure in the reviewed studies was ED LOS (*N* = 28) [15–20, 22, 23, 25–28, 30–33, 35–42, 45–48], triage level (*N* = 1) [43, 44], ED resource usage (*N* = 1) [24], hospital admission (*N* = 1) [29], disposition from ED (*N* = 1) [21] and ED waiting room time (*n* = 1) [43, 44]. The twenty-three studies reported on the granularity of ED LOS in minutes [19, 32, 36, 39–41, 43, 44, 46, 47] or hours [18, 20, 22, 23, 25–28, 31, 33, 35, 38, 42, 48]. Some of these studies reported the mean or median of all patient ED stay. The mean of ED LOS ranged from 1 h to 9.2 days [15, 18, 33, 36, 37, 39–42, 44, 46] and the median of ED LOS ranged from 15 min to 54.6 h [20, 22, 27, 31, 33–35, 38, 43, 44, 46, 47]. Two studies did not provide a clear description of the statistical analysis methods [32, 45].

#### Candidate predictors

Not all studies reported on the predictor selection strategy. Table 2 shows the number and type of predictors in each model. Predictor variables were mostly measured at admission time or within the first 24 h of admission. Predictors selected for inclusion in modeling may have a large but spurious association with the outcome, which leads to predictor selection bias. Including such predictors increases the likelihood of over-fitting and thus over-optimistic predictions of a model’s performance for other individuals [49]. The number of continuous predictors was 0 [24, 36, 39, 41, 45, 47, 48] or 1 (age) [17, 21, 28, 35, 38, 40, 42–44, 46] or 2 [15, 20, 30] or 3 [26, 31] or 4 [33, 34] or 7 [29] or 8 [25] or 9 [19] or ten [27] or eleven [37] or eighteen [23]. The number of categories of all categorical predictors ranged from 0 to 19. Two studies used cut points to categorize continuous variables [20, 39]. Only one study used logarithmic transformation to transform the skewed continuous variables to approximately conform to normality [41].

As shown in Table 2, age, gender, acuity level, mode of arrival, patient disposition, and insurance type are important predictors for ED LOS that were used in most studies.

#### Sample size

The number of registered patients ranged from 100 [42] to over 4 million [16, 43] and the number of patients selected for model development or validation was between 42 [42] and 4,645,483 [16] patients.

#### Missing data

Most studies did not describe the completeness of data and/or handling of missing data. Some studies excluded all missing data for development and validation models. Ignoring the missing data can introduce bias. It is especially poor when the percentage of missing values per attribute varies considerably [23]. Differences between studies in the amount, type of missing data, and the methods used to handle this missing data may markedly influence model development and predictive performance. Only eight studies reported on the percentage of missing values [17, 21, 23, 28, 38, 42, 43, 47] and two studies described the handling of missing data [19, 22]. Specifically, these studies excluded all missing data for development and validation models.

#### Model development

Twenty-nine studies developed one or more new models for predicting emergency department LOS [24–33, 35, 38, 39, 42, 43, 45, 48]. Models were developed using Logistic Regression [15, 18, 20, 21, 23–26, 29, 31, 35, 38, 39, 45, 48], Artificial Neural Network (ANN) [15, 16, 22, 37, 45], convolutional neural networks (CNN) [15], generative adversarial network (GAN) [19], accelerated failure time (AFT) [34], time series [48], Gradient Boosting Machine (GBM) [32], Coxian phase-type distribution model [33], Decision tree algorithm [28], linear regression [21, 30, 36, 43], Poisson regression [27, 36], and various machine learning methods (Random Forest (RF), Support Vector Machines (SVM), Gradient Boosting (GB), AdaBoost, K-Nearest Neighbours (KNN), CatBoost, XGBoost, Decision Tree, Naïve Bayes) [15, 17, 18, 22, 23]. Note that these papers have used some of these machine learning models. It should be noted that only one study used the quantile regression analysis since the distribution of the response variable (ED service completion) was highly skewed, with long right tails [43].

Eight studies evaluated univariate associations with a prolonged LOS [24, 25, 27–29, 32, 35, 36]. Three studies used all candidate variables. The remaining studies did not mention how the initial set of variables was selected. Further details are shown in Table 2. Also, Table 4 shows the factors analyzed and statistics of the selected studies for this systematic review.

**Table 4:** Factors analysed and statistics of the selected studies for the systematic review

N	Author	Type of ED	Study Group (n)	Methods	Factors Analysed
1	Lee S [16]	Adult	4,645,483	Artificial Neural Network (ANN)	Age, sex, ECI, insurance, alive
2	Zeleke AJ [17]	General	12,858	Random Forest (RF), Support Vector Machines (SVM), Gradient Boosting (GB), AdaBoost, K-Nearest Neighbors (KNN), and Logistic Regression (LR)	Gender, age, mode of arrival, triage categories, specialty, problems
3	Lee H [18]	Adult	968	C Logistic Regression, gradient boosting machine (GBM), Naïve Bayes	Triage level, sex, age, visit day, visit type, referral, severe disease, emergency operation, admission type, retransfer, consultation, diagnosis, disease
4	Kadri F [19]	Pediatric	44,676	generative adversarial network (GAN)	Arrival date/time, age, sex, diagnostic, biology, echo, radiology, scanner, LOS
5	Lee K.S [20]	General	657,622	Logistic Regression	age, sex, insurance, injury code, ambulance attendance, transferred-in, date and time, initial triage, ventilation, diagnosis codes, Charlson comorbidity index (CCI), discharge status
6	Srivastava S [21]	General	33,727	Logistic Regression	age, gender, insurance, hospital type, patient location, admission month, encounter cost, comorbidities
7	Etu EE [22]	General, Covid-19	3,301	Logistic Regression (LR), gradient boosting (GB), decision tree (DT), random forest (RF)	Age, sex, race, covid symptoms, comorbidities, vital signs
8	Chang YH [23]	Adult	92,528	Random Forest (RF), Logistic Regression (LR), decision tree (DT), CatBoost, XGBoost	Age, sex, BMI, vital signs, consciousness, tracheotomy, transferred, arrival mode, bed request, comorbidity, pregnancy, complaints, LOS
9	d'Etienne JP [24]	Trauma	110,471	Logistic Regression, discrete event simulation	Age, sex, marital status, Ethnicity, Transfer mode, vital signs, Pox, complaint, ESI, ED crowding, Disposition
10	Laher AE [25]	Adult	11,383	Logistic Regression	Age, sex, race, HIV status, vital signs, laboratory results,
11	Bacchi S [15]	General	313	Artificial Neural Network (ANN), Random Forest (RF), convolutional neural network (CNN)	-
12	Sweeny A [26]	Geriatric	16,791	Multivariate regression	Age, sex, mode of arrival, day, time, triage type, arrival time, discharge destination
13	Sricharoen P [21]	General	504	Poisson regression	Age, sex, race, conditions, NYHA class, vital signs
14	Rahman MA [28]	Trauma	80,512	Data mining	visit type, Age, Gender Indigenous status, arrival, postcode, Triage category, problem, diagnostic, Day of week, Admit to ward, Mental health, referral, Consult, examination, Mental health request, Month, Hour
15	Curiati PK [29]	Geriatric	5,025	Logistic Regressions	Age, sex, No. of medications, diagnosis, fall, Hospitalization in the previous 6 m
16	Chen C-H [30]	General	12,962	Natural Language Processing (NLP)	Age, sex, BMI, Vital signs, arrival time, Taiwan triage scale, LOS
17	Street, M [31]	Geriatric	33,926	Logistic Regressions	Age, sex, language, marital status, hospital, day of arrival, arrival overnight, arrival mode, triage type, time to visit, imaging
18	Gill, S. D [32]	General	17,644	gradient boosting machine (GBM)	Age, sex, mode of arrival, referral, clinician group
19	Zhu, T [33]	General	894	Coxian phase-type (PH) distribution	Region, age, sex, arrival mode, arrival time, ESI, treatment area, admission date/time
20	Chaou C-H [34]	General	106,206	accelerated failure time (AFT)	LOS, triage to physician, age, sex, triage level, transferred, patient entity, daily ED consus
21	Warren M [35]	Psychiatric	6,335	multivariate Regressions	Age, sex,race,insurance, arrival mode, diagnosis, disposition, arrival hour, visit day, visit month
22	Prisk D [36]	Trauma	80,214	Poisson regression	Age, ethnicity, Socioeconomic deprivation, practitioner type, disposition, complaint, Australasian triage scale category
23	Launay CP [37]	Geriatric	993	Artificial Neural Network (ANN)	Age, sex, drugs, falls history, Temporal disorientation, home service, Acute organ failure, home living, diagnosis, LOS
24	Stephens R [38]	General	2,447	Logistic Regressions	Age, diagnosis, complaints, LOS, sex, insurance, triage day, disposition, disposition day, severity, transport to ED, unit type, Race
25	Casalino E [39]	General	20,845	multivariate Regressions	Age, ED disposition, sex, arrival type, acuity level, ED outcome
26	Green N [40]	Pediatric	780	Statistics methods	acuity level, disposition, LOS, number of resources
27	van der Linden C [41]	General	48,397	Statistics methods	-
28	Nejtek V. A [42]	Psychiatric	42	Categorical regression	Age, sex, race, marital status, insurance, clinical diagnoses, pharmacotherapy
29	Ding R [43]	General	48,896–58,316	Quantile Regression	date and time of registration; bed placement, initial contact physician, disposition decision, ED discharge, disposition status, inpatient medicine bed occupancy rate, age, sex, insurance status, and mode of arrival, acuity level and chief complaint
30	Chi, C. H [44]	General, Pediatric, Trauma	3,172	Statistics methods	Age, Sex, shifts, disposition, ESI levels, setting, LOS
31	Walsh P [45]	General	119	Artificial Neural Network (ANN)	Age, vital signs
32	Tanabe P [46]	General	403	Statistics methods	-
33	Jimenez, J [47]	General	32,758	Statistics methods	time to triage, triage duration, patients without visit by a physician, waiting time
34	Tandberg D [48]	Trauma	87,354	Time series	-

*ECI* Elixhauser Comorbidity Index cluster

#### Model performance measures

Fourteen studies reported calibration measures (i.e. the agreement between predictions and observed outcomes) among which six studies used the Hosmer–Lemeshow goodness-of-fit test [17, 18, 31, 34, 35, 39, 48], two studies used the visual inspection of the observed vs. predicted proportions [31, 43], five studies used the mean squared error [15, 17, 19, 30, 31], one study used the life-table method [34], two studies used calibration plots [17, 29], one study used the kappa statistic [45], and one study used the linear regression method to inspect the association of forecasts with the actual outcomes [48]. A total of 13 studies used the Receiver Operating Characteristic (ROC) curve to quantify the discrimination power of the prediction model (i.e. the ability of the model to discriminate between those with and those without the event) [15–18, 22–24, 29, 31, 32, 35, 37, 39]. Nine studies also calculated the sensitivity, specificity, and positive and negative predictive values [15–18, 22, 23, 29, 31, 37]. Note that limited use of the popular performance measures prevents us from integrating the prediction powers of the models.

#### Model evaluation

Among development studies, sixteen studies performed internal validation, which useda subset of the training dataset to estimate the model performance (*N* = 9 split sample and *N* = 7 cross-validation) [15–19, 22–24, 28–32, 43, 45, 48], three studies used the entire dataset for both training and evaluating the model [34, 35, 39], and twelve studies performed no evaluation approach [20, 21, 25, 26, 33–37, 40, 44, 47]. All six external validation studies assessed the predictive validity of the previously published models by investigating the relationship between scores and ED LOS, mostly using the correlation coefficients.

Emergency severity index (ESI), Canadian Emergency Department Triage and Acuity Scale (CTAS), Charlson comorbidity index (CCI), Korean Triage and Acuity Scale (KTAS), Pronto Atendimento Geriátrico Especializado (ProAGE) and Emergency Nurse Practitioners (ENPs) were six triage instruments that were validated by nine studies to assess these instruments in predicting ED LOS, hospital admission, and number of resources utilized. The results of these studies showed that there was an excellent correlation between the ESI (version 3&4), CTAS, and ENP-streaming and patients’ injury severity. The findings of these studies showed that mean LOS was significantly shorter for the patients in the ENP stream in comparison with their counterparts [41]. The mean of LOS in ED was also significantly higher for the patients with higher acuity levels in comparison with the patients with lower acuity levels (257 vs. 143, *P* < 0.001) [40]. Moreover, the patients with ESI 4–5 and 2–3 had the shortest and longest LOS in ED, respectively [44, 46].

#### Reporting on the developed model

All studies that developed a new model (*n* = 29) reported the final model. However, since it was not possible to provide a comprehensible representation of the ANN model, only the relative importance of each variable was estimated by counting the number of times each variable was selected as one of the top five variables by each NN in the ensemble. An ensemble is a 'committee' of neural networks that usually outperforms single neural networks. [45]. Six studies reported the regression coefficients [22, 29, 30, 38, 39, 43] and eleven studies were reproducible, since the final model, initial predictors, and final set of variables included in the model were reported [16–19, 22, 23, 28, 29, 34, 39, 45, 48].

#### Interpretation and discussion of the eligible studies

All studies presented the intended use and interpretation of the validated or developed model(s). Use intentions were mostly as a patient triage or risk management [9, 24–31, 33, 34, 45, 48], ED resource utilization [24, 25, 30, 44, 46–48], identifying patients suitable for treatment [41], and determining valid factors that are significant predictors for hospital/ED admission and ED LOS [26, 27, 29, 31, 32, 38–40, 42, 43]. All reviewed models were discussed based on the validation results of the studies. However, only five development studies [26, 28, 29, 45, 48] and three validation studies [41, 44, 46] have discussed the strengths and weaknesses of the models.

#### Reporting and methodological quality assessment score

Table 1 shows domains and (key) items of the used CHARMS [15] checklist accompanied with the reporting and methodological scores used for quality assessment of the studies. The highest possible reporting scores for the development and validation studies were 67 and 43 respectively. The total score per reporting item ranged from 0 to 68 which is the sum of the reporting score [0, 1, 2] over models. The highest methodological score was 8 for development studies and 6 for validation studies. The total score achieved per methodological item (the sum of the methodological scores [0, 1, 2] over models) ranged from 0 to 68.

## Discussion

The average length of stay is an increasingly concerning issue and an important index for bed administration, patient care, and consequently benchmarking of the emergency departments. Accurate prediction of LOS in ED will help physicians make informed decisions during risk assessment and patient stratification. This study aimed to quantify the methodological and reporting quality of prediction models which have been developed or externally evaluated to predict the LOS in ED.

The most important finding of this study is the remarkable differences in methods used for model development, different thresholds used to categorize the dependent variable, and inclusion of different patient groups which affected the comparability of the models. A total of 34 studies were published from 1994 to 2023 aiming to develop (*N* = 29) or externally validate (*N* = 5) the prediction models for LOS in ED. Different modeling approaches were used to generate the function predicting the outcome. Since the linear regression method is not applicable when the normality assumption is violated, about %44 of the development studies dichotomized the dependent variable using different thresholds and applied the Logistic Regression method. Five studies used different machine learning techniques to predict ED LOS. Of these, Gradient Boosting (GB) in two studies and CATBoost and generative adversarial network (GAN) in two other studies had the best results in predicting LOS [17, 19, 22, 23]. In one study Logistic Regression shows better results than machine learning methods [18]. In addition, Logistic Regression still had similar results compared to machine learning approaches.

Two studies used the Coxian phase-type distribution method and quantile regression because the response variable was highly skewed to the left [33, 40]. These methods seemed to be useful because, in the emergency setting, we need to make a serious investigation not only on the middle of the distribution but also on extreme events. ANN was also used in five studies [15, 16, 22, 37, 45]. Using different types of ANN, multilayer perceptron (MLP) had significant results than another type of ANN [37]. It has the advantage over Logistic Regression when the relationships between the inputs and the outputs are not straightforwardly expressed in a pre-specified parametric model. However, the lack of model specification and proneness to over-fitting makes it difficult to be used in clinical and administrative judgments. Tandberg et al. used time series analysis [35]. This approach can be useful when data are repeatedly measured over time. Gill et al. reported that they used the GBM method because it allows for modeling of interactions and nonlinearities within the data and can handle a large number of variables [33]. One study used a decision tree. This method can demonstrate important patterns intuitively, helping the clinician to make sense of potentially complex combinations of factors [28].

About 40% and 33% of the studies reported calibration and discrimination measures for categorized outcomes, respectively. The Hosmer–Lemeshow goodness-of-fit test was the most frequently used test to assess the agreement between predicted probabilities and observed outcomes for categorized outcomes. However, this widely used test has several drawbacks (e.g., poor interpretation and limited power). Moreover, the ROC curve which is the most popular method to evaluate the discrimination power of the prediction models with binary variables was only used in thirteen studies among which only nine studies calculated the classification-based performance measures (e.g., sensitivity, specificity, etc.). There are numerous traditional and novel performance measures for estimating the prediction power of the models [54] which have been rarely used in both development and evaluation studies.

Patient triage and resource optimization was the most mentioned intention of the model in the included studies. Triage is commonly used to rapidly identify the patients who require immediate care and the patients who cannot wait before being evaluated and treated. Once the LOS is precisely predicted, the physicians will perform an informed and accurate risk assessment and consequently patient stratification. This will also result in helping optimize the bed occupation rate as well as resource utilization in crowded Eds [55].

Both development and validation studies completely reported the following key items: number and type of predictors, definition of the candidate predictors, time of predictor measurement, number of participants and outcomes/events, and event/(binary) variable ratio, model interpretation, source of data, and sample size.

### Limitations and strengths

A strength of our study is that we systematically assessed the studies found by a framework published by Moons et al. (CHARMS) [14] extended with additional items from other studies that developed a prediction model [12, 56, 57] to assess the studies and models on reporting and methodological quality. We included studies that developed prediction models for ED LOS and did not include studies that evaluate whether a specific characteristic influences or is a predictor for ED LOS. Another strength is that this is the first systematic review of ED LOS prediction models for emergency department patients.

Our study has some limitations over previous reviews of prediction models for LOS in emergency departments. First, there exist some prediction models developed for patients with ED LOS which do not meet our inclusion criteria because they partly addressed the prediction of ED LOS. Second, there is possible some papers are missed in our review. Third, we limited our research to English-language articles. Fourth, we researched only one database, PubMed. Our research terms may not have revealed all aspects of the topic.

### Implications for clinicians/policymakers/researchers/model developers

Available prediction models for LOS in ED have poor to fair levels of methodological and reporting quality which makes them barely useful for clinical practice and administrative decision making. Many important issues are required to be addressed to provide accurate predictions of the LOS in ED.

### Future research

We recommend that all development and validation studies use a clear definition of LOS in ED. This might be considered as an essential prerequisite for the comparability of the models. Moreover, models that have not been validated may not perform well in practice because of deficiencies in the development methods or because the new sample is too different from the original. Thus, it is highly recommended to evaluate available models on different datasets and update them if required. It should be noted that using the Transparent Reporting of a multivariable prediction model for Individual Prognosis Or Diagnosis (TRIPOD) checklist can help future investigators to improve the reporting quality and indirectly the methodological quality of prediction model studies.

## Conclusion

Various studies on prediction models for ED LOS were published but they are fairly heterogeneous and suffer from methodological and reporting issues. Model development studies were associated with a poor to a fair level of methodological quality in terms of the predictor selection approach, the sample size, reproducibility of the results, missing imputation technique, and avoiding dichotomizing continuous variables. Moreover, it is recommended that future investigators use the confirmed checklist to improve the quality of reporting. Physicians considering using these models to predict ED LOS should interpret them with reservation until a validation study using recent local data has shown that they obtain moderate calibration and produce accurate predictions.

## Data Availability

The data that support the findings of this study is available by corresponding author upon request.

## Abbreviations

ED: Emergency Department
ED LOS: Emergency Department Length of Stay
ANN: Artificial Neural Network
ESI: Emergency Severity Index
CTAS: Canadian Emergency Department Triage and Acuity Scale
PRISMA: Preferred Reporting Items for Systematic Reviews and Meta-Analyses
CHARMS: Critical Appraisal and Data Extraction for Systematic Reviews of Prediction Modelling Studies
CNN: Convolutional Neural Networks
GAN: Generative Adversarial Network
GBM: Gradient Boosting Machine
DT: Decision tree
LR: Logistic Regression
KNN: K-Nearest Neighbours
AFT: Accelerated Failure Time
NYHA: New York Heart Association

## Footnotes

1 https://www.rayyan.ai/
